# Prediction and Estimation of River Velocity Based on GAN and Multifeature Fusion

**DOI:** 10.1155/2022/7316133

**Published:** 2022-08-21

**Authors:** Yan Wang, Weiwei Chen, Yulan Wang

**Affiliations:** ^1^School of Artificial Intelligence, Xi'an Aeronautical Polytechnic Institute, Xi'an 710089, China; ^2^School of Information Engineering, Chang'an University, Xi'an 710064, China; ^3^Department of Mathematics and Physics, Hebei University of Architecture, Zhangjiakou 075000, China

## Abstract

The necessity of predicting and estimating river velocity motivates the development of a prediction method based on GAN image enhancement and multifeature fusion. In this method, in order to improve the image quality of river velocity, GAN network is used to enhance the image, so as to improve the integrity of image data set. In order to improve the accuracy of prediction, the image is extracted and fused with multiple features, and the extracted multiple features are taken as the input of CNN, so as to improve the prediction accuracy of convolution neural network. The results show that when the velocity is 0.25 m/s, 0.50 m/s, and 0.75 m/s, the accuracy of improved method can reach 85%, 90%, and 92%, which are higher than SVM, VGG-16, and BPNET algorithms. The above results indicate that the improvement has certain positive value and practical application value.

## 1. Related Work

China has abundant water resources, but it is also threatened by catastrophic flooding. However, due to the limitations of field environmental factors and the increase of data volume, using the traditional manual measurement for river velocity not only has great constraints, but also faces serious personnel threats. With the maturity of modern image acquisition technology and the wide application of artificial intelligence algorithm, how to use noncontact equipment for river monitoring has become the focus of current thinking and research.

It is found that the texture features of river channel image have a certain mapping relationship with the flow velocity, but the features are relatively single. If the traditional contact real-time monitoring is used, its accuracy will be greatly affected. To solve this problem, Tauro F et al. proposed a nonlinear learning fluid prediction method, so as to provide reference for fluid velocity prediction [1]. Gholami et al. [2] proposed a new CRBFNN model for the problem of river flow velocity. Through the model, the river flow velocity of 60° and 90° was predicted. The results showed that the model was more suitable for the prediction of bend flow velocity of 60^o^ [2]. Khuntia et al. [3] proposed to use multivariate regression to analyze and predict the river velocity and found that the machine learning algorithm performed better than the traditional Shiono and Knight methods [3]. Based on particle tracking velocimetry, Eltner et al. [4] collected and estimated River images. The results show that the deviation of this method is between 4% and 5% [4]; Zhang et al. [5] used the reflection technology of BeiDou system to inverse the river velocity and then obtained the river velocity with small error [5].

All the above studies focus on fluid velocity prediction, but its accuracy needs to be further improved. In view of the above problems, some scholars also proposed to process the collected water flow images. For example, Gulrajani et al. [6] proposed to introduce gradient punishment mechanism into the WGAN algorithm to better improve the WGAN algorithm [5]. David proposed an image processing technology based on the BEGAN algorithm to solve the contradiction between picture type and generation [6].

The above research shows that this paper proposes a flow image processing and velocity prediction method based on GAN algorithm and CNN algorithm, which takes the Yellow River flow image as the object. Meanwhile, the feasibility of the construction method is verified.

## 2. The Basis of Convolutional Neural Network Algorithm

**Figure 1:**
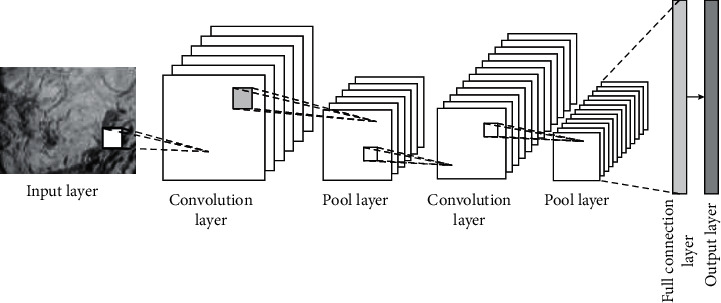
Structure of CNN.

Convolutional neural network has been widely used in classification problems. The characteristics of the convolutional neural network are reflected in translation invariance, feature extraction, and so on. Moreover, it has the characteristics of weight sharing and local connection. The structure is divided into multiple layers, including input, output, and convolution layer. And each layer has different functions. In the different layer structure, the calculation methods are different to some extent. The basic structure of the network is shown in Figure 1.

The convolution layer needs to carry out convolution processing for the upper feature maps or input information. In this process, some learnable convolution kernel and activation function should be used to obtain the corresponding output results. The formula is shown below [7–9]:

(1) $$\begin{array}{c} x_{j}^{l}=f\left(u_{j}^{l}\right),\\ u_{j}^{l}=\sum_{i\in M_{j}}x_{j}^{l-1}*k_{ij}^{l}+b_{j}^{l}, \end{array}$$

where *l* represents the convolution layer; *f*(·) represents the activation function of *l*; *x*_*j*_^*l*^ represents the output of channel *j* in *l*; *u*_*j*_^*l*^ represents the net activation of channel *j* in *l*, and, specifically, it is obtained by the convolution layer *l*-1 of the output feature map with *l* convolution and then adding *b*_*j*_^*l*^. *k*_*ij*_^*l*^ represents the convolution kernel weight matrix, whose size is generally 1*∗*1, 3*∗*3, 5*∗*5, etc. *M*_*j*_ represents the set of input feature maps.

The pooling layer is divided into two types: maximum pooling and average pooling, which is divided according to the input feature graph. Then, the dividing matrix block pixels are processed by sliding window. This layer is also called the downsampling layer, and the formula is shown as follows [10–12]:

(2) $$\begin{array}{c} x_{j}^{l}=f\left(u_{j}^{l}\right),\\ u_{j}^{l}=\beta_{j}^{l}\text{down}\left(x_{j}^{l-1}\right)+b_{j}^{l}. \end{array}$$

Here, down(·) represents the pooling function to realize the function of narrowing the input feature map; *β* represents the weight coefficient of pooling layer; *u*_*j*_^*l*^ represents net activation, which means that it is necessary to combine the previous layer output feature map with the current layer pooling function, and then add *b*_*j*_^*l*^.

For convolutional neural network, the features of input information can be extracted in the execution, specifically using the convolution and pooling repeated superposition. The convolution is divided into two parts: high level and low level. The former mainly extracts image semantic information, and so on. The latter extracts details, such as image edge information. Combined with the previous analysis, it can be seen that the characteristics of convolutional neural network include weight sharing and local connection, among which the weight sharing can effectively reduce the parameters, because the neurons weight on each feature mapping surface is consistent. The local connection means that the input of each neuron is connected with the local acceptance domain of the previous layer. In this way, the local features can be extracted. Meanwhile, the location relationship with other features can be clarified.

## 3. Flow Rate Classification Network Based on Multifeature Fusion

### 3.1. Multifeature Fusion

Traditional classification methods can achieve higher accuracy by deepening the network depth or broadening the network width of the classification model, namely, increasing the number of convolution layers or adding more feature map dimensions to the single convolution layer. However, the direct result of increasing the number of convolution layers is that the network parameters and computational complexity will greatly increase, and the training process will consume a lot of hardware and software resources. In addition, the increase of model layers can just improve the classification effect of the network within a certain range. When the number of layers increases to a certain depth, the gradient disappearance may occur during the backpropagation [13]. And the feedback information of shallow convolution cannot be obtained to adjust the weight size. Therefore, too deep structure will lead to performance degradation of the classification network. Taking the above considerations into account, this chapter proposes a multifeature fusion mechanism.

**Figure 2:**
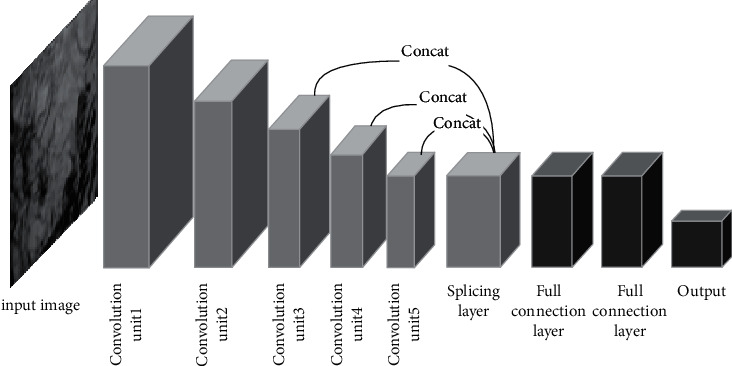
Diagram of multifeature fusion.

Figure 2 shows that, without increasing the network depth and spatial parameters, the learning robustness of the classification model for target data can be improved.

The feature maps of multiple convolution middle layers are fused. Under the normal use of data semantic information obtained by deep convolution, the local detail features reinforce information obtained by shallow convolution. The classification goal of higher accuracy can be achieved [14–17]. The calculation method is shown as follows:

(3) $$\begin{array}{c} L_{n}=\text{Concat}\left(L_{k},L_{p},L_{q}\right). \end{array}$$

It can be seen from the formula that *k*, *p*, *q* represent the number of convolution layers of fusion output; *L*_*n*_ represents the output feature map of the convolution layer *n*. The output of each convolution layer is multichannel feature map, and the size is the same. But the size is directly related to the number of convolution layers, which are negatively correlated. In the multiscale fusion, the size of the feature map needs to be adjusted first, which means that the nearest neighbor interpolation method is used for upsampling, and then splicing. So, the multilevel feature map fusion can be achieved.

### 3.2. Classification Network Structure

**Figure 3:**
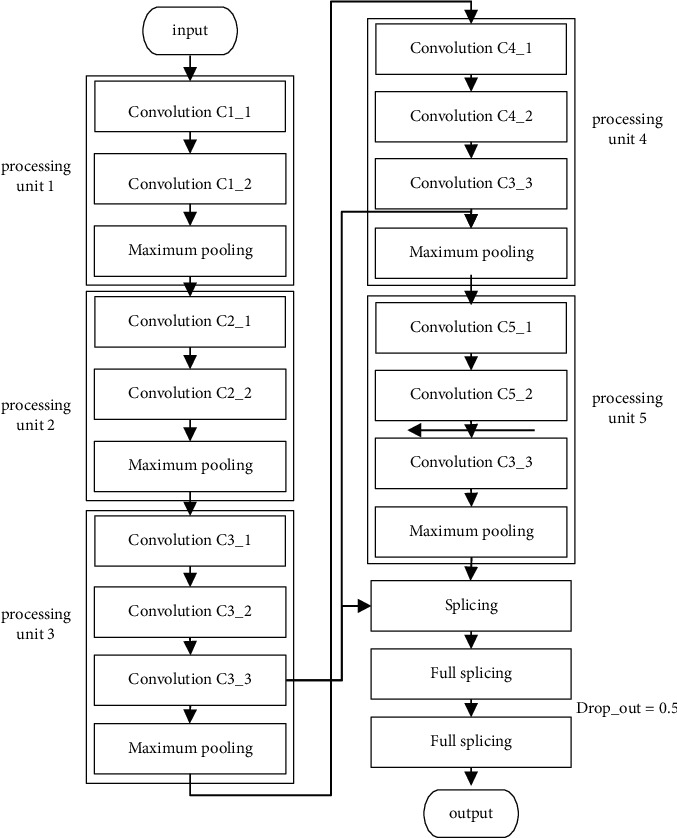
Classification network structure.

As shown in Figure 3, VGG-16 is adjusted and applied to image recognition. The classification model consists of one input layer and five convolutional processing units. Each unit contains two to three unequal convolution layers, a single pooling layer, two full connection layers, a Dropout layer, and a feature fusion layer [18–22]. The feature fusion mechanism is introduced to upsample the flow feature map output from convolution C5_2 layer. Then fuse it with the feature map output from convolution C3_3 layer and convolution C4_3 layer to form the feature fusion layer. In this network, the 3*∗*3 small convolution kernels are stacked to increase the depth of the model, and multiple Rectified Linear Units (ReLU) are added, which can reduce the computational complexity in the classification and recognition process. Apart from that, it can alleviate the overfitting problem to some extent. Before training the network, the multitype images generated by model learning in Chapter 2 and Chapter 3 are mixed with real samples. 70% of the data are randomly selected as the training set, and the rest as the test set. Dropout mechanism is added after the full connection layer of the classification network to alleviate the problem of overfitting during training.

The use of feature fusion mechanism has certain advantages, so that the underlying features on classification decision-making is strengthened. It is easy to identify the image with no significant difference in detail features. So, it has higher robustness. Even if the error between the training set and the test set is large, the higher recognition accuracy can still be achieved.

## 4. Experiment and Analysis

### 4.1. Experimental Subjects

**Figure 4:**
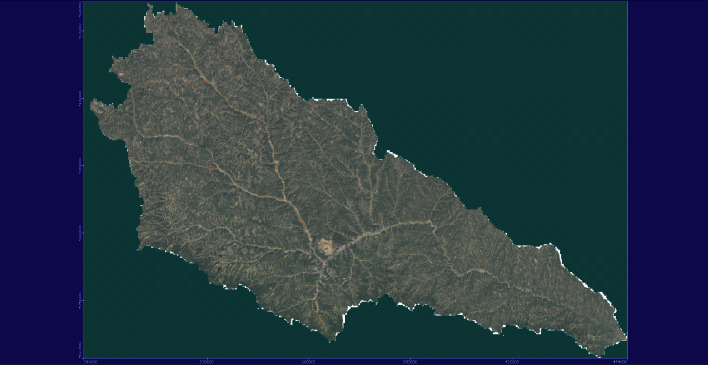
General River and tributary map.

In this paper, a section of the Yellow River is analyzed as an example. The precipitation in this section basically stays within 1800 to 2000 mm, and there are significant changes. The precipitation is mainly concentrated in summer, and there are many rainstorms. It is easy to cause mountain floods and other disasters. Therefore, it is necessary to monitor and analyze the flow velocity of each tributary in this region which is as follows in Figure 4.

### 4.2. Flow Image Acquisition

**Figure 5:**
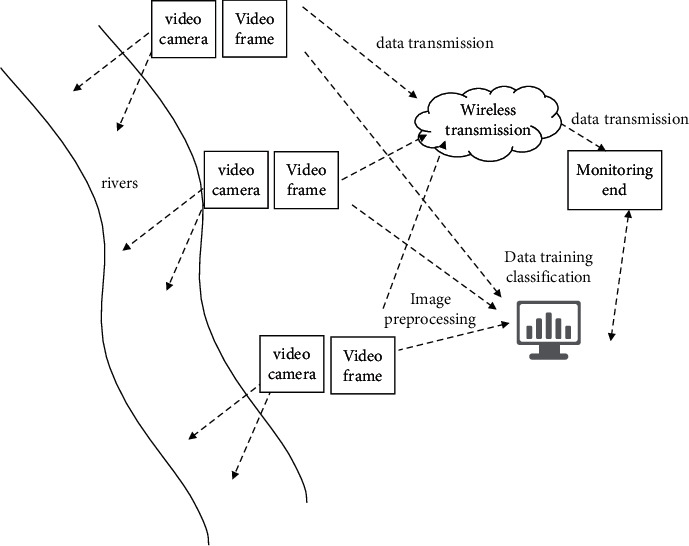
Water flow image acquisition scheme.

The professional camera devices are set up on both sides of boundary card river to collect water images. And the collected images are transmitted through wireless equipment. Install cameras at an appropriate position in the building and set the frame rate to 60 fps at an appropriate spacing. In addition to capturing images of the river, information such as weather and velocity values should also be recorded. The specific solution is shown in Figure 5.

Many modules are involved in the river image acquisition, including video acquisition and wireless transmission module. The image acquisition and transmission functions are completed as a result of each module's effective cooperation. In the video acquisition module, the function camera with high performance is used to collect images according to the set parameters. The data transmission module realizes the video transmission function, which adopts the Unicom CDMA network. In addition to the above part, it also includes the central monitoring platform. The monitoring terminal can use different access methods. First is to automatically record and update the monitoring position of the image, and second is to query the current record of the image.

During image acquisition, not only the current average velocity is recorded, but also the velocity at both sides of the same time and middle positions is compared, so as to meet the requirements of hierarchical estimation. After the collection is completed, the professional software is used for video interception. So, the training sets can be obtained, totaling 8000 pieces, 1000 pieces for each power station. The classification interval is set, then the flow rate images were clipped. Therefore, the images with the same flow rate are classified based on the flow rate label. The flow rate resolution designed in this study is 0.5 m/s, which is totally divided into five flow rate intervals.

### 4.3. Experimental Platform Configuration

The experimental platform is set up, and the computing platform uses the Dawn W580-G20 server, Nvidia Tesla k8/0 computing card, dual processor architecture, and 480 GB/S video memory bandwidth. The computing performance is higher, which meets the requirements of this paper.

### 4.4. Image Processing

In order to enhance the completeness of data and improve the robustness of Figure 3 network, it is proposed to process the collected water flow image data before prediction. Based on GAN and current models, the relatively real pseudo samples can be obtained. But there are some problems with GAN. First, the diversity of sample generation cannot be guaranteed. Second, the training process is difficult [23, 24]. Third, the discriminator is stronger than the generator in the initial stage, so it is difficult for them to achieve a balance in the adversarial training. To solve the problem, David et al. designed a boundary-balanced generative adversarial network. In this network, a new loss function is set up and an autoencoder is introduced. Meanwhile, the GAN is able to learn the texture features directly from raw data, without modeling. Thus, it is consistent with the real data to the maximum extent available. However, as the BEGAN described earlier did not set generator constraints, the fitting effect is affected to some extent. In this study, the above problems are analyzed, and a method based on conditional boundary balance generative adversarial network is designed. Conditional label information has been added to this method to guide the direction in which the data flow is generated. At the same time, the reliability of the generated image is verified to ensure that the generated image meets the higher quality requirements.

#### 4.4.1. Image Generation of Basic Label Information

To study the image generation strategy based on label information, firstly, the samples containing labels need to be extracted. Then, the conditional information is added in the boundary balance network to guide the image output based on category information. In the design, the label information *y* is added to the input of the generator and spliced with the sampled noise *z*. So, the corresponding category's images are generated under guidance. The basic form of the objective function is shown below [25]:

(4) $$\begin{array}{c} \underset{G}{\min}\max V\left(D,G\right)=E_{x∼p_{\text{dova}}}\left(x\right)\left[\log\;D\left(xly\right)\right]+E_{z∼p_{z}\left(z\right)}\left[\log\left(1-D\left(G\left(z|y\right)\right)\right)\right], \end{array}$$

where *D*(*x|y*) represents the probability of judging that the input image belongs to the real sample, while *D*(*G*(*x|y*)) represents the probability after the generator mapping. The loss function is as follows:

(5) $$\begin{array}{c} \text{Loss}\left(s\right)={\left|s-D\left(s\right)\right|}^{l},\;l\in 1,2. \end{array}$$

In the formula, *s* represents the sample through autoencoder, and *l* represents the *l*_1_ or *l*_2_ norm.

In the algorithm, the discriminator and generator have different functions, which can minimize the reconstruction errors of real image and generated image, respectively. Bulldozer distance (EM) is introduced to fit the loss distribution of the autoencoder. The specific formula is as follows:

(6) $$\begin{array}{c} W\left(\mu_{1},\mu_{2}\right)=\underset{\gamma \in \left(\mu_{1},\mu_{2}\right)}{\inf}E_{\left(x_{1},x_{2}\right)∼\gamma }\left[\left\|x_{1}-x_{2}\right\|\right], \end{array}$$

Here, *μ*_1_ and *μ*_2_ represent the loss distribution of real data and generated data respectively, while *W*(*μ*_1_, *μ*_2_) represents the set of all possible joint distributions of both of them.

#### 4.4.2. Generate Image Verification Mechanism

This part designs a verification module, which is added into *D* to identify the extracted image features. The input data is processed based on the nonlinear method and inputs the results in Softmax layer, where the probability of the category can be calculated. The probability formula of an output sample belonging to category *i* is shown as follows:

(7) $$\begin{array}{c} y_{i}=\frac{e^{a_{i}}}{\sum_{k=1}^{C}e^{a_{k}}},\;\forall i\in 1,\ldots ,C. \end{array}$$

It can be seen from the formula that *C* represents the number of categories to be predicted. The full connection layer output *a*_1_, *a*_2_, ..., *a*_*c*_ can get the corresponding probability distribution by the Softmax layer processing. The loss function is cross entropy, and the specific formula is as follows:

(8) $$\begin{array}{c} \text{Loss}=-\sum_{k=1}^{n}\sum_{i=1}^{C}t_{ki}\log\left(y_{ki}\right). \end{array}$$

The above formula revealed that *n* represents the number of samples; *k* stands for some sample; *i* stands for category; *t*_*ki*_ is the probability of *k* real categories; *y*_*ki*_ is the probability that the model pair *k* belongs to *i*.

#### 4.4.3. The Overall Model Construction

**Figure 6:**
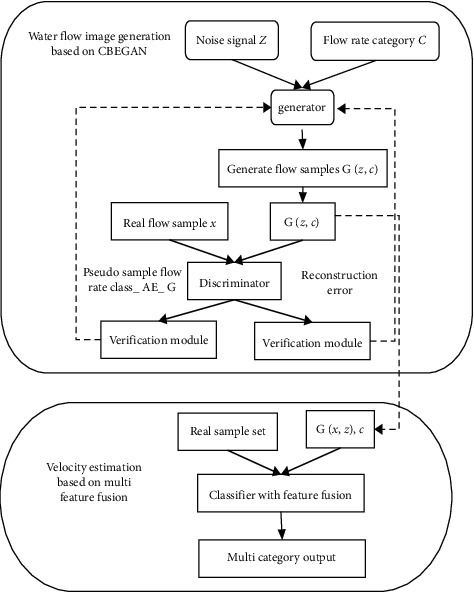
Proposed model based on GAN.

Combined with the above design, the flow velocity estimation model is constructed as shown in Figure 6.

where the whole model is divided into several parts, including three modules, namely, flow image generation, fogging, and velocity estimation. The design of each module is as follows:

  Step 1: Collect the video of water flow in this area, which can be divided into two weather conditions. The first is fog blocking, and the second is no blocking, namely, normal illumination. During recording, it is necessary to record the velocity on both sides and in the center of the river, and the flow rate should be determined. Except for the above operations, the appropriate cropping and other operations are required to support the subsequent processing.
  Step 2: First of all, the real sample is constructed, which means that the small flow data obtained in the previous step is corresponding to the flow velocity interval. Then, the discriminator and generator are constructed. Meanwhile, the uniform noise signals are input into the generator, and the mixed generated samples and real samples are input into the discriminator.
  Step 3: To verify the reliability of the generated flow image, in this process, the classifier with the same structure as the discriminator is used to analyze the flow velocity category. Hence, the optimal model weight is determined on this basis.
  Step 4: To generate the flow images, the key part of this step is the CBEGAN network.
  Step 5: To generate the data set, normalize the generated samples in the fourth and sixth steps, mix the generated samples and original samples in a random way, and then divide them into training set and test set, which account for 70% and 30%, respectively.
  Step 6: To build CNN, input the training set into the designed classification network. So, the recognized flow category can be obtained. Then the verification can be achieved through the test set. During the test, the input image is clipped with the same size, and the output results are weighted and averaged. Finally, the current time flow rate can be obtained.

### 4.5. Result Analysis

#### 4.5.1. Image Generation Quality Verification

**Figure 7:**
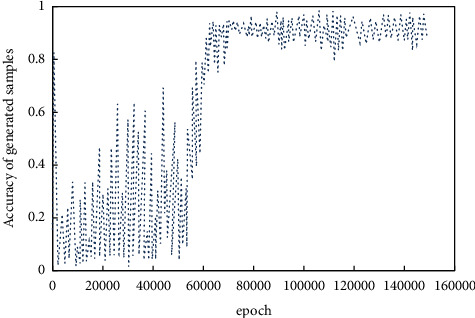
Accuracy of generated image.

**Figure 8:**
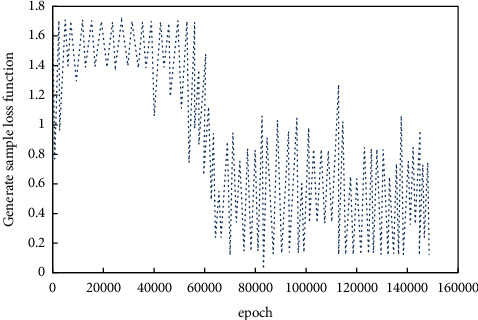
Loss function value of generated image.

*(1) Verification Mechanism Results Analysis*. The results of verification mechanism are analyzed, and the accuracy curve and category label loss function are shown in Figures 7 and 8 respectively. According to the information in the figure, the quality stability of generated images in the initial stage of training is low, which is related to the training imbalance. And it corresponds to a higher loss function value. When the iterations increase, the adversarial network becomes stable, and the probability of accurate classification increases gradually. For the average accuracy of 0.95, the probability of generating the corresponding category flow image is significantly improved after adding the label information.

**Figure 9:**
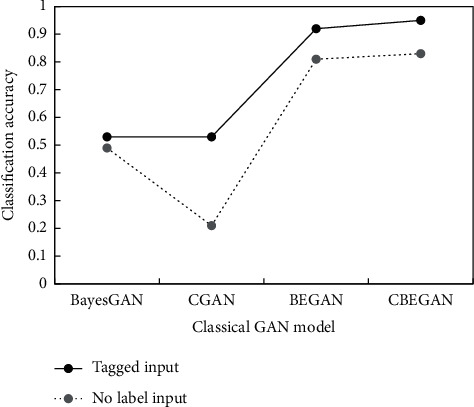
The effect of image generation under different models.

*(2) Image Generation Quality*. The results in Figure 9 show that the recognition accuracy of naive GAN and CGAN models is consistent, because the latter is the result of a slight improvement of the former. The classification accuracy obtained by the BEGAN model is significantly higher, because it improves the naive GAN greatly. However, the CBEGAN model designed a special validation module to constrain the flow of generated network data by the loss function. Thus, the higher accuracy is achieved relative to the BEGAN model.

It can be seen that CGAN is difficult to compute unlabeled data efficiently with unsupervised input. Compared with the naive GAN, the generation sample accuracy of BEGAN model is higher, which is related to the self-encoder that exists. The CBEGAN model designed in this study can transfer learning information to the model and then realize the adjustment of weight parameters.

Comparing the GAN network with conditional and unconditional input, it is found that the accuracy of the former generated sample is higher. It is mainly related to the mitigation of the freedom problem in training, which improves the quality of the generated sample.

*(3) Quality Analysis of Generated Image*. In image quality analysis, there are 5 different flow rate levels, namely, 0.5, 1, 1.5, 2, 2.5, and the unit is m/s. After the data set is established, it is calculated by the SIM indicator. The formula is as follows:

(9) $$\begin{array}{c} \text{SSIM}\left(x,y\right)=I\left(x,y\right)*c\left(x,y\right)*s\left(x,y\right), \end{array}$$

Here, *x* and *y* belong to two images, and *l*(*x*, *y*), *s*(*x*, *y*), and *c*(*x*, *y*) are the brightness, structural similarity, and contrast of images in turn.

**Figure 10:**
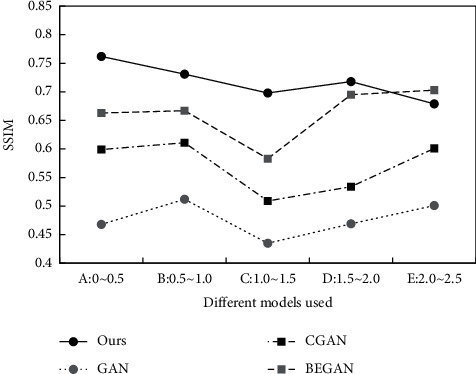
Comparison of different methods of SSIM.

The above indexes can be used to analyze the image distortion. If the index is large, it means that the quality of the generated image is higher. The SSIM calculation results of different methods are shown in Figure 10.

The information above shows that, compared with GAN, CGAN, and BEGAN, the structural similarity of the algorithm was improved by 0.25, 0.14, and 0.05, respectively. So, the improvement effect was significant compared with the two front algorithms. Compared with the third model, the labeled mechanism can also improve the quality of the generated image, which proves the feasibility of the proposed algorithm.

Here are two images of three-channel *X* and *Y*. The size of them is *m∗n*. The formula of MSE is as follows:

(10) $$\begin{array}{c} MSE=\frac{1}{m*n}{\sum_{k}^{3}\sum_{i=0}^{m-1}\sum_{j=0}^{n-1}\left(x\left(i,j,k\right)-y\left(i,j,k\right)\right)}^{2}. \end{array}$$

In the formula, *k* represents the number of channels.

In this study, the peak signal-to-noise ratio (PSNR) index is also introduced for evaluation, and its formula is as follows:

(11) $$\begin{array}{c} \text{PSNR}=10*\mathrm{lg}\left(\frac{{\text{MAX}}^{2}}{\text{MSE}}\right). \end{array}$$

**Figure 11:**
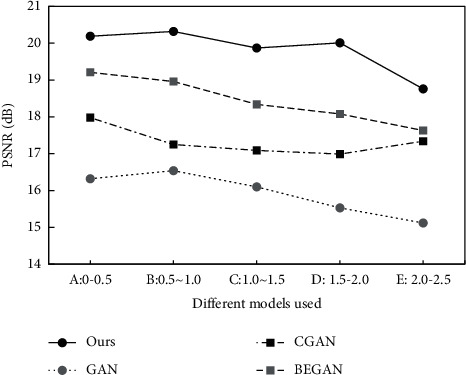
Comparison of different methods of PSNR.

The above formula shows that the MAX represents the maximum color value of image points, which is 255.  Figure 11 lists the calculation results of each method.

The results show that the PSNR index of CGAN is significantly higher than that of GAN, which verifies the positive role of the labeled mechanism. Compared with CGAN, the PSNR of BEGAN is improved again, which verified that improving loss function can improve the image quality.

Compared with other methods, the algorithm designed in this paper has some advantages in the peak signal-to-noise ratio, but the overall PSNR is low. It is presumed to be related to the image compression caused by the autoencoder. The relevant studies are needed to further improve the quality of the generated image.

#### 4.5.2. Analysis of Multifeature Fusion Effect

**Figure 12:**
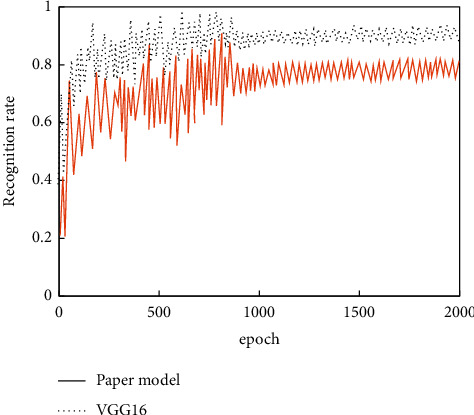
Recognition rate comparison of proposed model and VGG-16.

**Figure 13:**
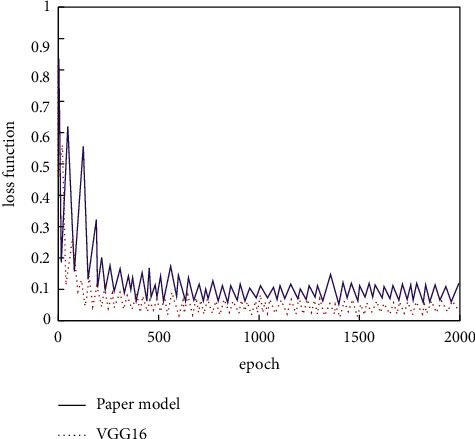
Loss function of proposed model and VGG-16.

The adversarial network generated samples are mixed with the real samples. Then the VGG-16 model is adjusted using the transfer learning method. The unsampling and other processing are carried out for the obtained feature maps. After adjusting to a consistent size, the deep semantic and shallow detail information is fused and unified input to the full connection layer. After completing the above operations, the feature fusion network is compared with the untreated network. The training data are uniformly normalized, and the iterative recognition rate and other information are recorded. The final results are shown in Figures 12and 13.

The information in Figure 11 shows that, compared with the unfused model, the fused model achieves higher recognition accuracy under the condition of the same iterations, which has basically converged around 800 times. Combined with the above analysis, it can be seen that the feature fusion method can be adopted for the feature recognition problem with insignificant difference, which is helpful to enhance the effect.

#### 4.5.3. Flow Rate Estimation Analysis in Natural State

**Figure 14:**
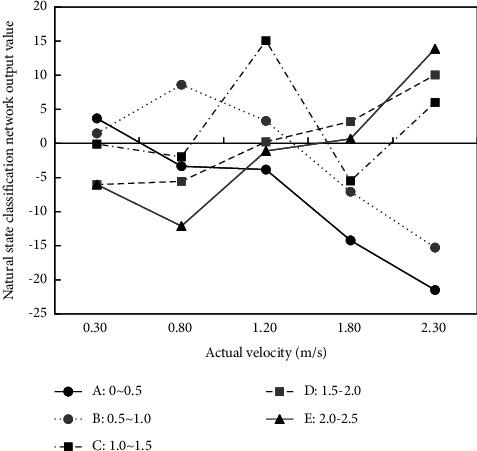
Output of model in normal state.

It is necessary to select appropriate test set data in natural state flow rate analysis. Here, the original *A*-*E* sample data are selected. In addition, the images without flow rate labels are used to uniformly input into the classification network for processing. The result is as shown in Figure 14. The information in the table shows that the predicted classification results are basically consistent with the real flow rate levels, which verifies the validity of the model.

Here, when different images are input, the higher the network output result is, the higher the probability of the category is. For the images with different flow velocity intervals, the differentiation is different. The differentiation of B and C images is higher, and the degree of *A* and *D* images is lower. For *E* image, it is found that the category similarity has a certain influence on the discrimination.

**Figure 15:**
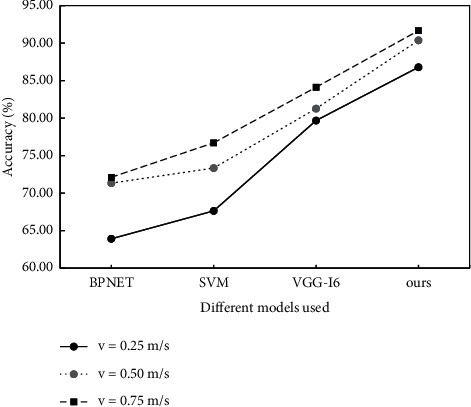
Comparison of recognition rate in normal state.

The difference of recognition accuracy is analyzed based on different methods, as shown in Figure 15. The same data set is used in the verification, and the velocity resolution of multiple groups, respectively, is set as 0.25, 0.5 and 0.75, and the unit is m/s.

## 5. Conclusion

In summary, it can be seen that the prediction of river flow image velocity can be effectively realized through the enhancement of river image and multifeature fusion combined with convolutional neural network. The results show that the method has obvious advantages, which is not only in the accuracy of speed prediction, but also in the loss function of the algorithm itself. Through the research, the problem of single feature of river image and the problem of traditional manual real-time acquisition are changed, and the information ability of river disaster early warning is greatly improved.

## Data Availability

The experimental data used to support the findings of this study are available from the corresponding author upon request.
